# Effects of Different Rearing Methods on the Intestinal Morphology, Intestinal Metabolites, and Gut Microbiota of Lueyang Black-Bone Chickens

**DOI:** 10.3390/ani15121758

**Published:** 2025-06-14

**Authors:** Shuang Zeng, Linqing Shao, Mingming Zhao, Ling Wang, Jia Cheng, Tao Zhang, Hongzhao Lu

**Affiliations:** 1 School of Biological Science and Engineering, Shaanxi University of Technology, Hanzhong 723001, China; 15310855926@163.com (S.Z.); slq010807@126.com (L.S.); 19913479179@163.com (M.Z.); wangling619@163.com (L.W.); chengjia@snut.edu.cn (J.C.); zhangtao780823@snut.edu.cn (T.Z.); 2 Engineering Research Center of Quality Improvement and Safety Control of Qinba Special Meat Products, Universities of Shaanxi, Hanzhong 723001, China; 3 Qinba State Key Laboratory of Biological Resources and Ecological Environment (Incubation), Hanzhong 723001, China

**Keywords:** Lueyang black-bone chicken, rearing method, intestinal morphology, intestinal metabolites, gut microbiota

## Abstract

The Lueyang black-bone chicken represents a distinct indigenous avian breed native to China and it is a slow-growing broiler breed. The gut, whose primary function is to digest food and absorb nutrients, is also home to a large and diverse microbial community. The intestinal morphology, intestinal metabolites, and gut microbiota are critical determinants of nutrient utilization efficiency and immune health in poultry. This study investigates the impact of two distinct rearing modalities—cage-raised (CR) and cage-free (CF)—on the intestinal morphology, intestinal metabolites, and gut microbiota of the duodenum and cecum in Lueyang black-bone chickens. Additionally, we have integrated metabolomics and microbiome analyses. Morphological assessments revealed that, in comparison to the CR group, the CF group exhibited a significant increase in duodenal villi height (VH) and crypt depth (CD) (*p* < 0.01). Furthermore, there was a notable increase in the number of intestinal inflammatory cells within the CF group. Non-targeted metabolomics indicated an upregulation of omega-3 series polyunsaturated fatty acids and bile acid metabolites in the CR group. Conversely, the CF group demonstrated significantly elevated levels of lysophosphatidylcholine (LPC) and phosphatidylcholine (PE) in the intestine. Microbiome analysis revealed that in the duodenum, beneficial bacteria (e.g., *Lactobacillus*) were the dominant genera in the CF group, while the *Bacteroides* predominate in the CR group. Correlation analyses indicated a positive association between LPC levels and the presence of eight bacterial genera, including *Ureaplasma*. The omega-3 series polyunsaturated fatty acids were positively correlated with three bacterial genera, such as *Flavobacterium*. Notably, bile acid metabolites exhibited a significant positive correlation with *Rikenellaceae_RC9_gut_group*. In conclusion, this study provides novel insights into how rearing methods influence intestinal morphology, intestinal metabolites, and gut microbiota, offering a new perspective for the scientific management of poultry with the premise of ensuring animal health and welfare.

## Simple Summary

The Lueyang black-bone chicken, a significant poultry resource in China, exhibits unique medicinal value and ecological adaptability. This study investigates the effects of cage-raised (CR) and cage-free (CF) rearing systems on intestinal morphology, intestinal metabolites, and gut microbiota of Lueyang black-bone chickens, thereby elucidating the critical relationship between rearing methods and gut health. In a comparative analysis, we observed that the CF group exhibited a significant increase in intestinal inflammatory cells, accompanied by increased levels of lysophosphatidylcholine (LPC) in the gut. Conversely, the CR group showed upregulation of omega-3 polyunsaturated fatty acids and bile acid metabolites, which have anti-inflammatory, cholesterol metabolism, and immune barrier enhancement effects. This investigation provides a scientific foundation for optimizing poultry farming by examining the metabolite–microbiota interaction network.

## 1. Introduction

With the improvement in living standards, consumers have increasingly high demands for the quality of meat and food safety. The production of high-quality meat products is closely related to the breed, feeding methods, and animal welfare. China’s indigenous chicken breeds exhibit a wealth of genetic diversity and unique characteristics, with evidence suggesting that the meat quality of these local chickens surpasses that of commercial varieties [1]. For instance, the Guangxi Partridge chicken, a renowned indigenous breed from southern China, exhibits firm meat texture due to the development of coarse muscle fibers [2]. Furthermore, the Gushi chicken breast is distinguished by its high unsaturated fatty acid content, which enhances the production of flavor compounds, such as alkanes, aldehydes, and ketones during cooking, thereby ensuring the meat’s stability and superior flavor [3]. Lueyang black-bone chickens, a slow-growing broiler breed, is the largest indigenous black-bone chicken breed in China. Black-bone chickens are considered to contain nutritional benefits but also exhibit medicinal properties [4]. Recent investigations have indicated that Chinese chicken breeds also exhibit significant advantages in terms of disease resistance and immune response. The Huai Xiang chicken, a well-known yellow-feathered slow-growing broiler breed, is predominantly raised in southern China due to its tolerance for roughage and robust disease resistance [5]. Moreover, studies have demonstrated that Beijing oil chickens possess strong immunity against avian influenza virus (Ab-AIV) and Newcastle disease virus (Ab-NDV) [6]. This resilience may be attributed to the traditional free-range rearing practices of local chicken breeds, which facilitate the development of meat quality and enhance disease resistance by providing a diverse diet, increased physical activity, and a complex growth environment.

Recent research has demonstrated that variations in rearing practices have a direct impact on the diversity of gut microbiota in animals. Muyyarikkandy MS et al. identified significant differences in the gut microbiota between commercial chickens and those raised in a backyard. The predominant phylum in the gut microbiome of commercial chicken was found to be *Firmicutes*, whereas the backyard chicken exhibited higher levels of *Bacteroidetes* and *Proteobacteria* [7]. Additionally, chickens raised in ground-farmed environments are exposed to a greater variety of microorganisms through their behavior of pecking at litter and excreta, resulting in a more diverse intestinal microbiota compared to caged chickens, particularly in the duodenum, jejunum, and ileum [8]. It has been observed that the population of *lactobacilli* in the colons of chickens raised on organic farms is lower than that in those from conventional farms, leading to an increased proportion of *Firmicutes* and *Bacteroidetes* [9]. Additionally, Li et al. compared the intestinal microbes and metabolites of Lueyang black-boned chickens in cage and free-range mode and found that the rearing method significantly affected the composition and function of intestinal microbes. Specifically, the relative abundance of *Firmicutes* in the intestinal tract of chickens in the free-range group was found to be lower than that of the cage group, whereas the relative abundance of *Bacteroidetes* was higher in the free-range group. Consequently, the ratio of *Firmicutes* to *Bacteroidetes* was significantly reduced in the free-range group compared to the cage group [10]. In conclusion, diverse rearing methods alter the diversity and composition of intestinal microbiota by influencing the dietary intake, environmental conditions, and physiological states of the animals.

The diversity of gut microbiota serves as a critical indicator of intestinal health in animals, with research indicating that such diversity can significantly influence disease resistance in these organisms [11]. For example, the gut microbiota can engage in direct interactions with the host’s immune system by stimulating plasma cells to produce immunoglobulin A (IgA), facilitating the maturation and migration of dendritic cells, activating effector T cells, and modulating T and Th17 cell responses, thereby bolstering immune defenses [12,13]. Meanwhile, the gut microbiota can also exert indirect effects on host health through its metabolic byproducts [14]. As an illustration, *Bacteroides* species are capable of generating acetic acid and propionic acid by degrading the host’s mucus polysaccharides and dietary components [15]. These metabolic activities subsequently promote the differentiation of regulatory T cells and enhance the production of interleukin-10 (IL-10), which is essential for the maintenance of intestinal homeostasis [16,17]. Additionally, probiotics have been shown to positively influence the gut health of animals. Research has indicated that *Lactobacillus* significantly increases the population of goblet cells in the small intestine and jejunum, thereby improving intestinal barrier function [18]; *bifidobacteria* enhance immune responses by producing butyrate, which directly augments the mucus-secreting capacity of intestinal goblet cells and supports the integrity of the intestinal mucus layer [19].

In summary, rearing methods can affect animals’ gut health in various ways, especially through the gut microbiota. The Lueyang black-bone chicken, an indigenous chicken breed originating from Lueyang County, Shaanxi Province, China, exhibits distinct characteristics compared to other local breeds. The meat quality of the Lueyang black-bone chicken is renowned for its unique flavor profile and rich nutritional content [20]. Furthermore, its meat is abundant in various antioxidant peptides [21], thereby exhibiting medicinal potential in the prevention of atherosclerosis, diabetes, neurodegenerative diseases, and the retardation of aging [22]. Prior investigations have predominantly emphasized its meat quality attributes, while the impact of rearing methods on the gut health of this poultry remains relatively understudied. Consequently, this study took cage-raised and cage-free Lueyang black-bone chickens as the object, aiming to explore the effects of rearing methods on the intestinal morphology, intestinal metabolites, and gut microbiota to provide a theoretical foundation for the development of the Lueyang black-bone chicken industry and the optimization of scientifically based husbandry practices.

## 2. Materials and Methods

### 2.1. Ethics Approval Statement

All animal experimental procedures in this study were approved and implemented following the guidelines formulated by the Animal Care and Utilization Committee of Shaanxi University of Technology (NoSLGQD/09/2017).

### 2.2. Animal Resources and Feeding Program

This experiment was carried out at the Shaanxi Longjia Agricultural Technology Co., Ltd. (Hanzhong, China) broiler breeder farm. Before the experiment, a total of 240 one-day-old female healthy Lueyang black-bone chicks were raised in cages within the same brood house and were reared to 30 days old. After the basic immunization protocol, 240 hens were randomly assigned into 2 groups (the cage-raised and cage-free) with 6 replicates per group (20 chickens per replicate). The CR group adopted a three-layer three-dimensional single cage (28 cm× 30 cm × 50 cm), and every chicken had an average floor area of 0.15 m^2^. The cage system’s average temperature was 20–25 °C, the relative humidity was 65–70%, and the indoor photoperiod was a 16:8 light/dark cycle. In the CF group, the poultry was fed on an indoor ground (bird/0.15 m^2^). Additionally, a hillside forested area (120 m^2^) was provided adjacent to the indoor facility, offering an average of 1 m^2^ per bird. The environmental conditions within the indoor environment were controlled similarly to those of the CR group. During the experimental period, the outdoor ambient temperature in the surrounding mountainous forest region ranges from 20–25 °C. The architectural design features windowed structures, with the poultry house constructed on a cement floor. The free-range poultry housing and the adjacent forested activity zone were interconnected, permitting unrestricted, voluntary movement of the poultry within the area. Both rearing methods provided unrestricted feed and water for the chickens. A starter diet was used for the first 30 days, and then replaced with a grower diet [4]. The dietary formulations and nutrient levels were consistent across both groups.

### 2.3. Sample Collection

At 3 months of age, 12 chickens from each group were randomly selected and subsequently euthanized by carbon dioxide anesthesia and cervical dislocation. Following the dissection, tissue samples from the duodenum and cecum were promptly collected. The intestinal samples from each section were fixed in 4% paraformaldehyde until analyzed. Each intestinal segment was embedded in paraffin. To analyze intestinal microbiota, the duodenal and cecal contents from each group were scraped and placed in sterilized 1.5 mL centrifuge tubes. These samples were subsequently stored at −80 °C after being rapidly frozen in liquid nitrogen.

### 2.4. Histological Analysis

Duodenal and cecal tissue morphology was assessed by HE staining. The intestinal samples fixed in 4% paraformaldehyde were subjected to secondary sampling and then placed in an automatic dehydrator for gradient alcohol dehydration, xylene clearing, and wax infiltration before embedding. The prepared paraffin sections were dewaxed with water by gradient and subsequently stained. The intestinal tissue structure of each group was observed with an Olympus BX53 upright microscope (OLYMPUS Corporation, Tokyo, Japan), the villus length (VL) and crypt depth (CD) of the duodenum were measured, and the VL/CD ratio was calculated. Three sections were selected for determination, and the average value was calculated. The villus height (VH) measures the vertical distance from the apex of the villus to the opening of the crypt, while the crypt depth (CD) is the distance from the base of the villus to the crypt opening.

### 2.5. Nontargeted LC-MS Metabolomics Analysis

First, 50 ± 5 mg of intestinal samples were accurately weighed into a 2 mL centrifuge tube, and 400 μL of extraction solution (methanol/water = 4:1 (*v*/*v*)) containing 0.02 mg/mL of internal standard (L-2-chlorophenylalanine) was added for metabolite extraction. In addition, 20 μL of the supernatant was pipetted and mixed as a quality control sample. In this experiment, the mass spectrometric data were collected using a Thermo UHPLC-Q Exactive HF-X Mass Spectrometer (Thermo Fisher Scientific, Waltham, MA, USA) for LC-MS analysis [23]. Metabolite information was obtained by matching MS and MS/MS mass spectrometry information with metabolic data in the HMDB (http://www.hmdb.ca/ (accessed on 6 March 2025)) and Major’s libraries. The preprocessed data matrix was subjected to principal component analysis (PCA) and orthogonal least partial squares discriminant analysis (OPLS-DA) to the preprocessed data matrix using the R software package “ropls” (Version 1.6.2). The selection of significantly different metabolites was determined based on the variable weight value (VIP) obtained from the OPLS-DA model and the *p*-value of the Student’s *t*-test. Metabolite identification and pathway analysis were then performed using Metaboanalyst (http://www.metaboanalyst.ca (accessed on 6 March 2025)) and the Kyoto Encyclopedia of Genes and Genomes (KEGG) database (http://www.genome.jp/kegg/ (accessed on 6 March 2025)) according to standard procedures. Unique metabolites were also screened, and dynamic heatmaps were generated by the OmicShare website tool. Correlation analysis employs the MajorBio cloud platform to analyze relationships between differential metabolites and the differential microbiota, thereby assessing the strength of the association between these two variables.

### 2.6. Intestinal Microbiome Analysis

DNA of the microbial communities was extracted from the duodenal and cecal samples according to the instructions of the FastPure Stool DNA Isolation Kit (Magnetic beads) (MJYH, Shanghai, China). Bacterial 16S rRNA (V3–V4 region) was amplified by PCR with primer pairs 338F (5′-ACTCCTACGGGAGGCAGCAG-3′) and 806R (5′-GGACTACHVGGGTWTCTAAT-3′). The paired-end raw sequencing sequences were controlled by fastp software (version 0.19.6) and spliced by FLASH software (version 1.2.11). Based on the ASVs’ information, the rarefaction curves, alpha diversity indices, Chao1 richness, Shannon index, and Good’s coverage were calculated with Mothur software (version1.30.2) [24]. The linear discriminant analysis (LDA) effect size (LEfSe) [25] (http://huttenhower.sph.harvard.edu/LEfSe (accessed on 7 March 2025)) was performed to identify the significantly abundant taxa (phylum to genera) of bacteria among the different groups (LDA score > 4, *p* < 0.05). All data analysis was performed on the Mega Biotech Cloud Platform (https://cloud.majorbio.com (accessed on 7 March 2025)).

### 2.7. Statistical Analysis

All experimental data analysis was performed using SPSS software (version 26.0), and graphs were generated using GraphPad Prism 8.0 software. The Shapiro–Wilk test was used to evaluate the normality of the data. On this basis, an independent samples *t*-test was conducted to compare the differences between the two groups, and *p* < 0.05 indicated a significant difference (*), *p* < 0.01 indicated a highly significant difference (**), and *p* < 0.001 indicated an extremely significant difference (***).

## 3. Results and Discussion

### 3.1. Effects of Different Rearing Modes on the Intestinal Morphology of Lueyang Black-Bone Chickens

**Figure 1:**
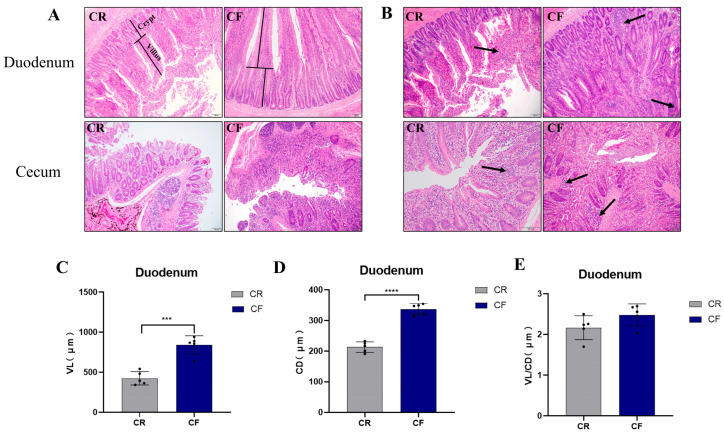
Effects of different rearing patterns on the intestinal morphology of chicken. (**A**,**B**) Histological observation of the duodenum and cecum. Black arrows represent inflammatory cell infiltration. Scale bar = 100 μm. (**C**) Duodenal villi length. (**D**) Duodenal crypt depth. (**E**) Villi–crypt ratio. *** *p* < 0.001, **** *p* < 0.0001. CR, cage-raised; CF, cage-free.

To explore the impact of feeding methods on the intestinal morphology of Lueyang black-bone chickens, histological sections were subjected to HE staining. The morphological characteristics of the duodenum and cecum (in micrometers) of the chickens are illustrated in Figure 1A,B. The findings indicate that the duodenal and cecal structures of Lueyang black-bone chickens in both the CR group and the CF group were intact and well defined, with no significant differences observed (Figure 1A). Compared to the CR group, the number of inflammatory cells in the CF group was increased, and the inflammatory infiltration was more obvious in the duodenum and cecum (indicated by black arrows in Figure 1B). Furthermore, by measuring the height of the intestinal villi (VH) and crypt depth (CD) and calculating the VH/CD ratio, it was found that the CF group exhibited a significant increase in both the VH and CD compared to the CR group (*p* < 0.01), but the VH/CD ratio did not show a significant change (Figure 1C,E). The above results suggest that the intestinal absorptive area in the CF group was enhanced, and the digestion and absorption of nutrients may have been stronger.

### 3.2. Untargeted Metabolomics Analysis

#### 3.2.1. Quality Control of Metabolomes Under Different Rearing Methods

**Figure 2:**
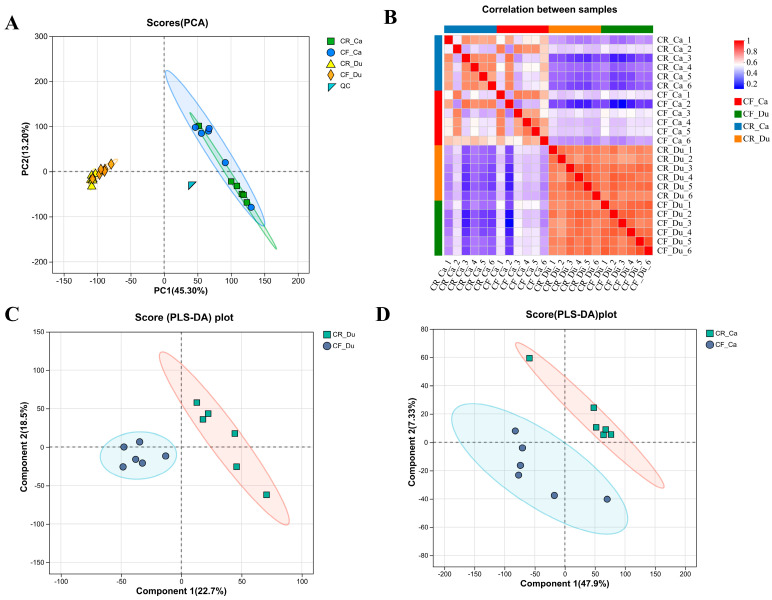
Multivariate analysis of metabolomics data from different rearing methods. (**A**) Sample PCA analysis; (**B**) sample correlation heat map; (**C**) PLS-DA analysis of duodenal samples; (**D**) PLS-DA sample analysis of cecal samples.

To investigate the factors contributing to variations in intestinal morphology associated with different rearing methods, a metabolomic analysis utilizing LC-MS detection technology was conducted. The results show that the QC samples in the PCA score plot are closely clustered around the coordinate origin, suggesting good experimental reproducibility. However, some cross-overlap was observed between the samples from the CR and CF groups in both the duodenum and cecum (Figure 2A). The sample correlation heatmap reveals distinct differences in metabolite profiles between the CR and CF groups (Figure 2B). PLS-DA demonstrated significant differences in the intestinal metabolites between the two groups in both the duodenum and cecum (Figure 2C,D). Quality control assessments confirmed that the different feeding methods influenced the intestinal metabolites of Lueyang black-bone chickens, thereby warranting further analysis of metabolite expression.

#### 3.2.2. Analysis of Differential Metabolites

##### Screening and Identification of Differential Metabolites

**Figure 3:**
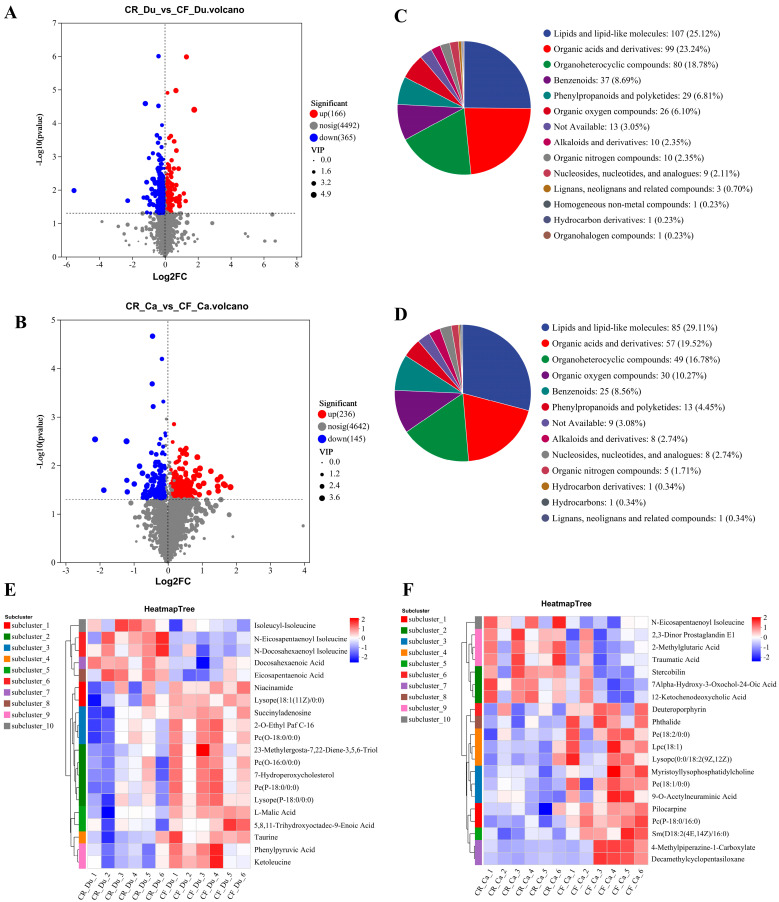
Screening and identification of differential metabolites under different rearing modes. (**A**) CR vs. CF volcano diagram in the duodenum; (**B**) CR vs. CF volcano diagram in the cecum; (**C**) classification of CR vs. CF DMs in the duodenum; (**D**) classification of CR vs. CF DMs in the cecum; (**E**) the 20 DMs in the duodenum; (**F**) the 20 DMs in the cecum.

Differential metabolite (DM) screening was conducted using thresholds of VIP > 1 and *p* < 0.05, with the results illustrated in volcano plots. In the duodenum, a total of 531 DMs were identified between the CR and CF, comprising 166 metabolites that were upregulated and 365 that were downregulated (Figure 3A). In the cecum, 381 DMs were detected between the CR and CF, with 236 metabolites upregulated and 145 downregulated (Figure 3B). The identified DMs were subsequently classified and characterized using the HMBD database. In the duodenum, the primary categories of DMs between the CR and CF included lipids and lipid molecules (25.12%), organic acids and their derivatives (23.24%), organic heterocyclic compounds (18.78%), and benzene ring compounds (8.69%) (Figure 3C). In the cecum, the DMs primarily consisted of lipids and lipid molecules (29.11%), organic acids and their derivatives (19.52%), organic heterocyclic compounds (16.78%), and organic oxygen compounds (10.27%) (Figure 3D). These findings indicate that the DMs in both the duodenum and cecum associated with the two rearing methods predominantly comprised lipid substances and organic acids. Lipids and lipid molecules are typically linked to energy metabolism and the structural integrity of cell membranes within intestinal metabolism, while organic acids and their derivatives are generated by gut microbiota during metabolic processes and are closely associated with intestinal health and immune functionality.

##### Cluster Analysis of Differential Metabolites

To visualize the trends of the two groups of DMs, the top 20 DMs were subjected to clustering analysis and separated into six distinct clusters. In the duodenum, the expression levels of omega-3 series polyunsaturated fatty acids, specifically docosahexaenoic acid (DHA) and eicosapentaenoic acid (EPA), were found to be upregulated in the CR group. In contrast, the CF group exhibited elevated levels of lysophosphatidylcholine (LPC) and lysophosphatidylethanolamine (PE), including Lysope(18:1(11Z)/0:0), 2-O-Ethyl Paf C-16, Pc(O-18:0/0:0), Pc(O-16:0/0:0), Pe(P-18:0/0:0), and Lysope(P-18:0/0:0). Furthermore, the expressions of 5,8,11-Trihydroxyoctadec-9-Enoic Acid, Niacinamide, L-Malic Acid, taurine, Phenylpyruvate, and ketoleucine were also significantly increased in the CF group (Figure 3E). LPC has a variety of biological functions, and it has been demonstrated that LPC can promote the release of inflammatory factors, such as interleukin (IL-6), which can compromise the integrity of the epithelial barrier, thereby intensifying the inflammatory response [26]. PE is an important component of cell membranes, and its synthesis and accumulation increase during cell membrane reorganization and repair [27]. Niacinamide, a water-soluble vitamin, plays a key role in the body’s energy metabolism pathways [28]. Nicotinamide, through decreasing the synthesis of tumor necrosis factor-α (TNF-α) and interleukin-6 (IL-6) [29], exhibits anti-inflammatory properties. Meanwhile, nicotinamide can also affect intestinal immune homeostasis by remodeling the structure of intestinal microbiota and metabolites. For instance, supplementation with nicotinamide has been shown to enhance the abundance of *Akkermansia* and *Lactobacillus*, which contributes to the thickening of the intestinal mucus layer and the production of anti-inflammatory short-chain fatty acids (SCFAs) [30]. L-malic acid, taurine, phenylpyruvate, and ketoleucine belong to organic acids and their derivatives. These compounds are synthesized by the gut microbiota as a result of metabolic processes and are crucial in modulating intestinal immune responses. Taurine is an important amino acid and can improve the morphology and structure of intestinal villi and enhance intestinal barrier function by increasing the expression levels of intestinal barrier-related proteins, such as claudin-1, ZO-1, and occludin [31]. Eicosapentaenoic acid (EPA) and docosahexaenoic acid (DHA) are omega-3 polyunsaturated fatty acids found in oily fish and fish oil supplements. They exhibit hypolipidemic effects [32], promote fat metabolism [33], possess significant anti-inflammatory functions [34], and have the ability to modulate gut microbiota [35].

In the cecum, the expression levels of bile acid metabolites and isoleucine derivatives, including 12-ketocholic acid, 7α-hydroxy-3-oxochol-4-en-24-oic acid(7α-HOAd), and N-Eicosapentaenoyl isoleucine, were found to be upregulated in the CR group. As with the duodenal results, the CF group showed high levels of LPC and PE, such as Pc(P-18:0/16:0), Pe(18:1/0:0), Myristoyllysophosphatidylcholine, Lysopc(0:0/18:2(9Z,12Z)), Lpc(18:1), Pe(18:2/0:0). Additionally, compounds such as Decamethylcyclopentasiloxane, Pilocarpine, Phthalide, 9-O-Acetylneuraminic Acid, and Deuteroporphyrin were identified to be increased, alongside an elevation in deoxy porphyrin levels (Figure 3F). Sphingomyelin serves as a crucial component of biofilms, primarily consisting of sphingosine and either phosphocholine or phosphoethanolamine. It is extensively found in the intestinal environment, where it exerts physiological functions and maintains intestinal homeostasis by regulating lipid metabolism [36], maintaining intestinal barrier function [37], and exhibiting anti-inflammatory effects [38]. 12-Ketocholic Acid and 7Alpha-Hydroxy-3-Oxochol-4-En-24-Oic Acid belong to bile acids and their derivatives, with 12-Ketocholic Acid being a secondary bile acid, while 7Alpha-Hydroxy-3-Oxochol-4-En-24-Oic Acid is a derivative of 3-oxocholic acid. Bile acids are cholesterol-derived molecules involved in essential physiological processes, including nutrient absorption, glucose homeostasis, and the regulation of energy expenditure [39]. Furthermore, bile acids can affect the abundance and composition of the gut microbiota through their antimicrobial activity and affect gut barrier function through associated receptors [40]. N-Eicosapentaenoyl isoleucine is a fatty amide formed by the combination of EPA with isoleucine, and its primary function within the intestinal tract may be associated with the EPA function. Therefore, the differences in intestinal metabolites were caused by a multitude of factors, including environmental conditions, dietary intake, and the host’s physiological characteristics under caged and free-range conditions.

##### Enrichment Analysis of Differential Metabolites

**Figure 4:**
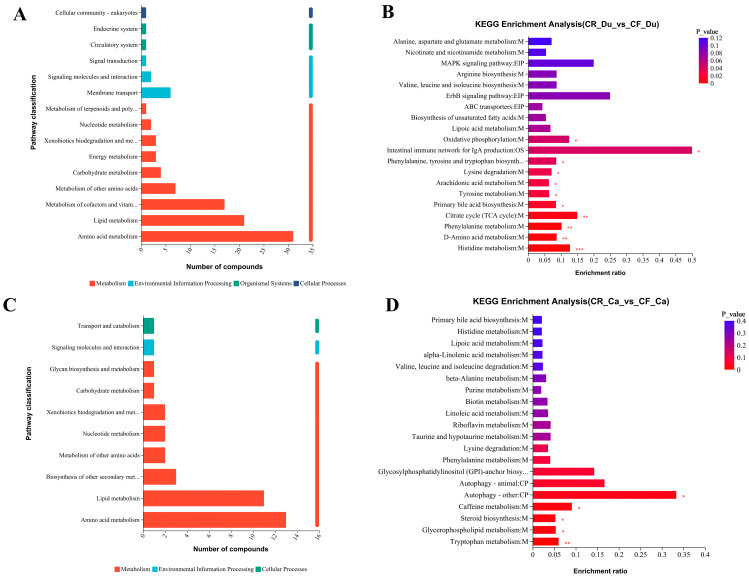
KEGG enrichment of DMs. (**A**) CR vs. CF DM functional pathways in the duodenum; (**B**) the enrichment of CR vs. CF DMs in the duodenum; (**C**) CR vs. CF DM functional pathways in the cecum; (**D**) the enrichment of CR vs. CF DMs in the cecum. * *p* < 0.05, ** *p* < 0.01, and *** *p* < 0.001.

KEGG functional pathway analysis was conducted on the selected metabolites to investigate the roles of the DMs identified between the two groups. Based on the KEGG database, 15 functional pathways were identified in the duodenum between the CR and CF groups. In the cecum, CR vs. CF identified 10 pathways. The data results show that in the duodenum, the DMs’ functional pathways primarily encompassed amino acid metabolism, lipid metabolism, glucose metabolism, etc. (Figure 4A). In the cecum, the DMs’ functional pathways mainly included amino acid metabolism, lipid metabolism, and biosynthesis of secondary metabolites (Figure 4C). The top 20 KEGG enrichment metabolic pathways are illustrated as follows. In the duodenum, the enrichment of DMs predominantly pertains to histidine metabolism, D-amino acid metabolism, and primary bile acid biosynthesis, as well as the biosynthesis of tyrosine and tryptophan (Figure 4B). In the cecum, the KEGG enrichment of DMs was mainly enriched in tryptophan metabolism, glycerophospholipid metabolism, steroid biosynthesis, caffeine metabolism, etc. (Figure 4D). The findings presented above suggest that the differences in metabolites mainly affected amino acid metabolism and lipid metabolism under different rearing modes. Amino acid metabolism and lipid metabolism are intricately linked to the gut microbiota. These metabolic pathways mediate intestinal barrier function, immune response, and energy metabolism reprogramming by producing different compounds, such as bile acid metabolites, unsaturated fatty acids, long-chain fatty acids, tryptophan, etc., ultimately affecting the physiological state of the body.

#### 3.2.3. Unique Metabolites Analysis

##### Screening and Identification of Unique Metabolites

**Figure 5:**
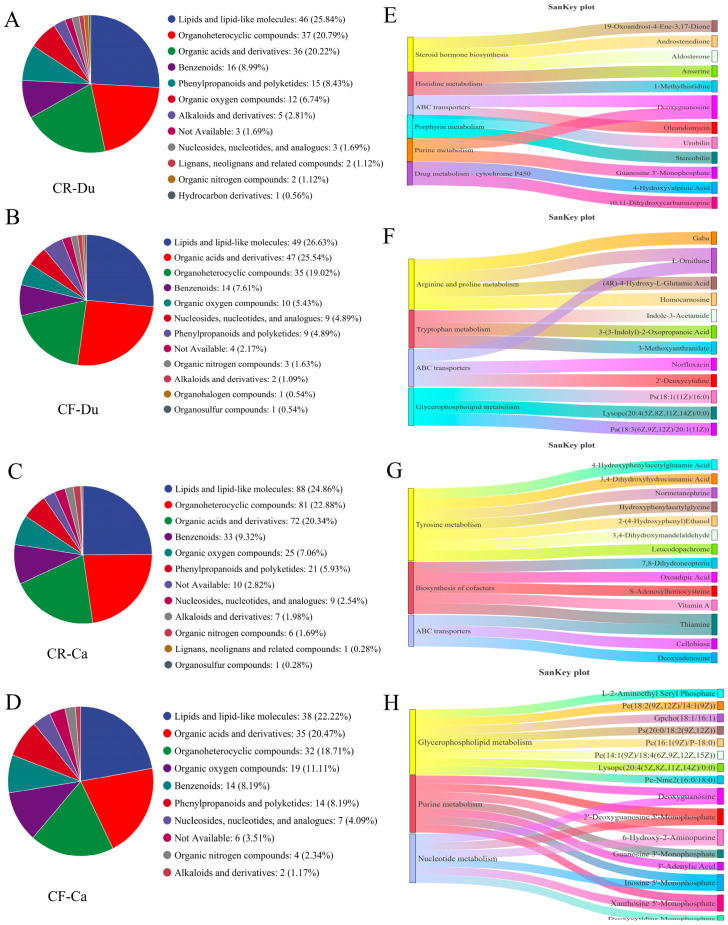
Analysis of unique metabolites under different rearing modes. (**A**,**E**) Classification and Sankey diagram of UMs from the CR group in the duodenum; (**B**,**F**) classification and Sankey diagram of UMs from the CF group in the duodenum; (**C**,**G**) classification and Sankey diagram of UMs from the CR group in the cecum; (**D**,**H**) Classification and Sankey diagram of UMs from the CF group in the cecum.

We also analyzed the unique metabolites of the two groups. The findings indicate that in the duodenum, the unique metabolites (UMs) associated with the CR group predominantly comprised lipids and lipid molecules (25.84%), organic heterocyclic compounds (20.79%), organic acids and their derivatives (20.22%), and benzene cyclic compounds (8.99%) (Figure 5A). The UMs in the CF group mainly included lipids and lipid molecules (26.63%), organic acids and their derivatives (25.54%), organic heterocyclic compounds (19.02%), and benzene ring compounds (7.61%) (Figure 5B). Compared with CR, the difference was the upregulation of organic acids and their derivatives in CF. Specifically, the concentrations of organic acids, including catechol, phenylacetic acid, and vanillic acid, were elevated in the feces of chickens raised in a cage-free environment. These organic acids have been shown to inhibit the proliferation of pathogenic bacteria, such as *Salmonella* and *Escherichia coli*, while simultaneously promoting the growth of beneficial microorganisms (lactic acid bacteria) by modulating intestinal pH. This regulation contributes to the enhancement of the intestinal microbial community structure [41].

In the cecum, the UMs identified in the CR group were primarily composed of lipids and lipid molecules (24.86%), organic heterocyclic compounds (22.88%), organic acids and their derivatives (20.34%), and benzene ring compounds (9.32%) (Figure 5C). The UMs of the CF group mainly included lipids and lipid molecules (22.22%), organic acids and their derivatives (20.47%), organic heterocyclic compounds (18.17%), and organic oxygen compounds (11.11%) (Figure 5D). Compared to caged environments, the content of organic heterocyclic compounds and organic oxygen compounds decreased, and the content of benzene ring compounds increased under free-range conditions. This suggests that various environmental factors under different rearing conditions influence the metabolism and accumulation of chemicals within the animal.

##### Function Analysis of Unique Metabolites

In the duodenum, CR-UMs were mainly enriched in steroid hormone biosynthesis, histidine metabolism, ABC transport, porphyrin metabolism, purine metabolism, and drug–cytochrome P450 (Figure 5E). The key compounds involved in steroid hormone biosynthesis included 19-Oxoandrost-4-Ene-3,17-Dione, Androstenedione, and Aldosterone. Anserine and 1-methylhistidine were the primary compounds involved in histidine metabolism. Furthermore, Deoxyguanosine and Oleandomycin were implicated in ABC transport. UMs in the CF group were primarily enriched in arginine and proline metabolism, tryptophan metabolism, ABC transport, and glycerophospholipid metabolism (Figure 5F). The compounds involved in arginine and proline metabolism included Gaba, L-Ornithine, (4R)-4-Hydroxy-L-Glutamic Acid, and Homocarnosine; the compounds involved in tryptophan metabolism included Indole-3-acetamide, 3-(3-Indolyl)-2-oxopropanoic acid, and 3-methoxyanthranilate; the compounds involved in ABC transport were Norfloxacin, 2’-Deoxycytidine, L-Ornithine; and the compounds involved in glycerol phospholipid metabolism were Ps (18:1(11Z)/16:0), Lysopc (20:4(5Z,8Z,11Z,14Z)/0:0), Pa(18:3(6Z,9Z,12Z)/20:1(11Z)). The metabolites under the cage conditions were mainly concentrated in steroid hormone biosynthesis. The interplay between steroid hormone biosynthesis and gut health is complex, encompassing several mechanisms. These include the influence of steroid hormones on the composition and functionality of the intestinal microbiota, their interaction with nuclear receptors to modulate gene expression, as well as their role in regulating the permeability of the intestinal immune barrier. For instance, the nuclear receptor glucocorticoid receptor (GR) can inhibit the NF-κB signaling pathway by binding to glucocorticoids, thereby diminishing the secretion of pro-inflammatory cytokines, such as IL-6 and TNF-α [42]. In addition, estrogens can enhance the expression of tight junction proteins (e.g., occludin) through ERβ and maintain intestinal barrier function [42]. Conversely, under CF conditions, metabolic pathways predominantly center on amino acid metabolism and glycerophospholipid metabolism, both of which play crucial roles in various physiological and pathological processes.

Within the cecum, the UMs of the CR group exhibited a notable enrichment linked to tyrosine biosynthesis, cofactor biosynthesis, and ABC transport. Compounds involved in tyrosine biosynthesis included 4-Hydroxyphenylacetylglutamic Acid, 3,4-Dihydroxyhydrocinnamic Acid, Normetanephrine, Hydroxyphenylacetylglycine, 2-(4-Hydroxyphenyl) Ethanol, 3,4-Dihydroxymandelaldehyde, and Leucodopachrome (Figure 5G). Cofactor biosynthesis encompasses 7,8-Dihydroneopterin, Oxoadipic Acid, S-Adenosylhomocysteine, Vitamin A, and Thiamine. Furthermore, Cellobiose, Deoxyadenosine, and Thiamine were implicated in ABC transport processes. The biosynthesis of tyrosine and the functionality of the intestinal immune system are interconnected through a sophisticated network encompassing metabolites, microbiota interactions, and enzymatic control. Tyrosine and its derivatives are crucial in preserving intestinal barrier integrity and modulating inflammatory responses. Tyrosine serves as a precursor for neurotransmitters, such as norepinephrine and dopamine. Norepinephrine can activate β-adrenergic receptors, thereby inhibiting the inflammatory response of macrophages and alleviating intestinal inflammation [43]. In addition, indole derivatives (e.g., indole lactate) produced by tyrosine metabolism are ligands for the aromatic hydrocarbon receptor (AhR). The activation of AhR induces the secretion of IL-22, which enhances intestinal epithelial barrier function through the STAT3 pathway and promotes the expression of antimicrobial peptides, such as β-defensin [44]. The UMs of the CF group are primarily enriched in glycerophospholipid metabolism, purine metabolism, and nucleotide metabolism. Compounds involved in glycerophospholipid metabolism include L-2-Aminoethyl Seryl Phosphate, PE(18:2(9Z,12Z)/14:1(9Z)), GPCho(18:1/16:1), PS(20:0/18:2(9Z,12Z)), PC(16:1(9Z)/P-18:0), PE(14:1(9Z)/18:4(6Z,9Z,12Z,15Z)), LysoPC(20:4(5Z,8Z,11Z,14Z)/0:0), and PE-NMe2(16:0/18:0) (Figure 5H). Compounds involved in purine metabolism biosynthesis include Deoxyguanosine, 2′-Deoxyguanosine 5′-Monophosphate, 6-Hydroxy-2-Aminopurine, Guanosine 3′-Monophosphate, 3′-Adenylic Acid, Inosine 5′-Monophosphate, and Xanthosine 5′-Monophosphate. Furthermore, Deoxyguanosine, Deoxycytidine Monophosphate, 2′-Deoxyguanosine 5′-Monophosphate, 6-Hydroxy-2-Aminopurine, Inosine 5′-Monophosphate, and Xanthosine 5′-Monophosphate are involved in nucleotide metabolism. These metabolic pathways collectively contribute to the construction and functional maintenance of cell membranes. Glycerophospholipids are major components of cell membranes, while purine and nucleotide metabolites, such as adenosine, participate in signal transduction [45,46,47]. Furthermore, these pathways play a crucial role in energy metabolism, with purine and nucleotide metabolism serving as the primary source of ATP, and fatty acids derived from glycerophospholipid degradation entering the β-oxidation pathway for energy production [48,49,50]. The results indicate significant differences in the gut-unique metabolites and their functional pathways among different rearing methods in the Lueyang black-bone chicken.

### 3.3. Effects of Different Rearing Modes on the Gut Microbiota of Chickens

#### 3.3.1. Analysis of Alpha Diversity of Gut Microbiota

**Figure 6:**
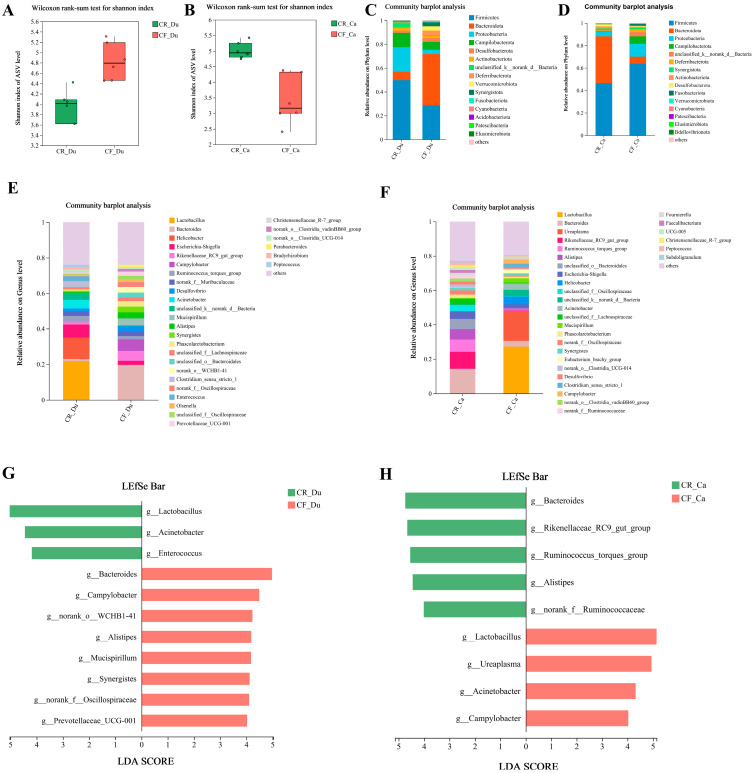
The effects of different rearing modes on the gut microbiota of chicken. (**A**) Alpha diversity of duodenal microbiota; (**B**) alpha diversity of cecal microbiota; (**C**) relative abundance of phylum in the duodenum; (**D**) relative abundance of phylum in the cecum; (**E**) relative abundance of genera in the duodenum; (**F**) relative abundance of genera in the cecum; (**G**) LEfSe plot of differential taxa in duodenum; (**H**) LEfSe plot of differential taxa in cecum.

The richness, diversity, and coverage of species in the community can be obtained through the alpha diversity index analysis. The results show that in the duodenum and cecum, the data were more dispersed in the CF group than in the CR group. Specifically, the duodenum exhibited higher species richness and diversity in the CF group. Conversely, the cecum displayed greater species richness and diversity in the CR group (Figure 6A,B).

#### 3.3.2. Analysis of Gut Microbiota Structure

This study investigated the impact of rearing methods on the gut microbiota composition of the Luyang black-bone chicken at the phylum and genus levels. The results indicate that in the duodenum, the dominant phyla in the CR group were *Firmicutes*, *Proteobacteria*, and *Campilobacterota*, while the CF group exhibited *Bacteroidota* and *Firmicutes* as the predominant phyla (Figure 6C). In the cecum, the CR group was primarily characterized by *Firmicutes* and *Bacteroidota*, whereas the CF group was dominated by *Firmicutes* and *Proteobacteria* (Figure 6D).

At the genus level, the gut microbiota of the CR group in the duodenum primarily comprised *Lactobacillus*, *Helicobacter*, *Escherichia-Shigella*, *Acinetobacter*, and *Ruminococcus torques group*. In contrast, the CF group’s gut microbiota mainly consisted of *Bacteroides*, *Rikenellaceae_RC9_gut_group*, *Campylobacter*, *Mucispirillum*, and *Alistipes* (Figure 6E). In the cecum, the gut microbiota of the CR group was dominated by *Bacteroides*, *Rikenellaceae_RC9_gut_group*, *Ruminococcus_torques_group*, *Alistipes*, and *unclassified_o__Bacteroidales*; whereas the CF group was primarily composed of *Lactobacillus*, *Ureaplasma*, *Helicobacter*, and *Acinetobacter* (Figure 6F). These findings suggest that different rearing methods impact the composition of the chicken gut microbiota.

#### 3.3.3. Differential Microbiota Screening

To systematically analyze the characteristic microbiota of the two rearing methods, the LEfSe species difference discrimination analysis was carried out on the microorganisms of the two groups. Linear discriminant analysis (LDA) with a threshold of four was used to screen out the intestinal microbiota with significant differences at the genus level between the two groups. In the duodenum, *Lactobacillus*, *Acinetobacter*, and *Enterococcus* were dominant in the CR group. Conversely, the CF group was characterized by the prevalence of *Bacteroides*, *Campylobacter*, *norank_o__WCHB1-41*, and *Alistipes* (Figure 6G). In the cecum, the CR group exhibited an increased relative abundance of *Bacteroides*, *Rikenellaceae_RC9_gut_group*, *Ruminococcus_torques_group*, *Alistipes*, and *norank_f_Oscillospiraceae*. The CF group showed an elevated relative abundance of *Lactobacillus*, *Ureaplasma*, *Acinetobacter*, and *Campylobacter* (Figure 6H). It is noteworthy that the abundance of *Lactobacillus* exhibited an inverse pattern across different intestinal segments between the two groups. It is hypothesized that the CR group, through optimized alimentation and environmental stability, facilitated the early colonization of *Lactobacillus* within the duodenum, whereas the CF group, predicated upon fiber fermentation and metabolic complementarity, may have locally enhanced *Lactobacillus* abundance within the cecum [51].

### 3.4. Correlation Analysis

**Figure 7:**
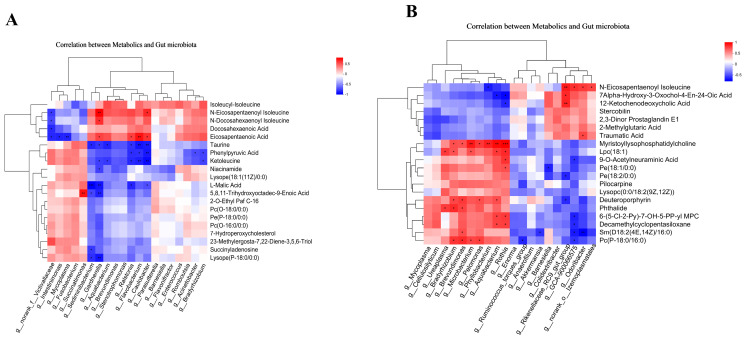
Correlations between gut differential metabolites and microorganisms under different rearing modes. (**A**) Correlation analysis in the duodenum; (**B**) correlation analysis in the cecum. * *p* < 0.05, ** *p* < 0.01, and *** *p* < 0.001.

To investigate the potential functional relationships between the gut microbiome and metabolites, a correlation analysis was performed to assess the degree of association between metabolite profiles and microbial abundance. This study focused on the top 20 differential microbiota and the top 20 metabolites for correlation analysis. In the duodenum, the analysis revealed a significant positive correlation between Succinatimonas and 5,8,11-Trihydroxyoctadec-9-Enoic Acid. Furthermore, Gardnerella exhibited positive correlations with 3 metabolites: eicosapentaenoic acid, N-Docosahexaenoyl isoleucine, and N-Eicosapentaenoyl isoleucine. Eicosapentaenoic acid demonstrated positive correlations with the gut microbiota genera Ralstonia, Flavobacterium, Caulobacter, and Gardnerella. In addition, Caulobacter exhibited a positive correlation with N-Eicosapentaenoyl isoleucine. Conversely, L-Malic Acid demonstrated a significant negative correlation with Sediminibacterium and Gardnerella. Moreover, Flavobacterium and Caulobacter exhibited significant negative correlations with both ketoleucine and taurine (Figure 7A).

In the cecum, Myristoyllysophosphatidylcholine exhibited a significant positive correlation with seven bacterial genera: Bradyrhizobium, Brevundimonas, Microbacterium, Pelomonas, Phyllobacterium, Aquabacterium, and Rothia. Lpc (18:1) correlated positively with five genera: Ureaplasma, Bradyrhizobium, Microbacterium, Aquabacterium, and Rothia. Furthermore, Pc(P-18:0/16:0) demonstrated a positive correlation with Bradyrhizobium, Brevundimonas, Microbacterium, and Pelomonas. In contrast, Rikenellaceae_RC9_gut_group showed a significant positive correlation with three metabolites: 12-Ketochenodeoxycholic Acid, 7Alpha-Hydroxy-3-Oxochol-4-En-24-Oic Acid, and N-Eicosapentaenoyl isoleucine (Figure 7B).

## 4. Discussion

The intestine acts as a congenital barrier for the stability of the internal environment of the body. It has an important impact on host physiology and pathology. The duodenum facilitates the breakdown of proteins, fats, and carbohydrates through the reception of digestive fluids from the pancreas, including pancreatic juice and bile, as well as gastric juices. The cecum, as the large intestine’s initial section, exhibits a high microbial density and serves as a critical region for microbial fermentation [52], thereby playing a unique physiological role. The surface of the small intestinal wall is characterized by villi, which are surrounded by crypt invaginations [53]. Research indicates a positive correlation between villus length and the number of immune cells [54]. Conversely, a decrease in villi length may lead to a disturbance in the distribution of immune cells, accompanied by inflammatory cell infiltration [55]. Crypt depth serves as a critical indicator of intestinal stem cell activity and cellular turnover. An increase in crypt depth is typically associated with enhanced stem cell proliferation [56]. For instance, deeper crypts often correlate with an increase in the number or activity of Paneth cells, leading to elevated α-defensin secretion and enhanced antimicrobial capacity [57]. Conversely, a reduction in defensins can predispose the gut to dysbiosis [58]. However, during bacterial or viral infections, the intestinal crypts may undergo excessive hyperplasia, thereby affecting goblet cell distribution, altering mucus composition, and decreasing the villus-to-crypt ratio, ultimately compromising barrier function [59]. In this study, the duodenal VH and CD in the CF group were significantly higher than those in the CR group, but the VH/CD values in the two groups did not change significantly. Furthermore, the CF group was accompanied by more obvious inflammation and infection. In free-range environments, animals encounter a diverse array of dietary sources while simultaneously facing elevated risks of pathogen exposure. This scenario leads to increased infiltration of inflammatory cells, consequently subjecting intestinal cells to heightened demands for repair and regeneration. In the duodenum, the predominant genera in the CF group, such as *Bacteroides* and *Rikenellaceae_RC9_gut_group*, can degrade dietary fiber via glycosidases and amylases, generating short-chain fatty acids (SCFAs), like acetate and propionate, which are crucial for the host’s energy supply and intestinal health. In conclusion, the multifaceted roles of *Bacteroides* and *Rikenellaceae_RC9_gut_group* in gut health may indirectly influence the maintenance and repair of intestinal villi and crypts.

The gut microbiota plays a central role in immune homeostasis through metabolites, immune cell regulation, and barrier maintenance. The findings of our study demonstrate that variations in rearing methods significantly impact the gut microbiome diversity in chickens. In the duodenum, the expression of *Lactobacillus*, *Acinetobacter*, and *Enterococcus* was upregulated in the CR group. Both *Lactobacillus* and *Enterococcus* belong to the phylum Firmicutes. It is widely acknowledged that *lactobacillus*, as one of the important probiotics in the animal intestine, can produce short-chain fatty acids (SCFAs). These SCFAs play a crucial role in promoting nutrient absorption [60], maintaining gut microbiota balance [61], enhancing immune function [62], and improving intestinal barrier function [63], thereby positively impacting animal gut health. *Enterococcus* is a Gram-positive, facultatively anaerobic genus of bacteria that is ubiquitously distributed in natural environments (e.g., soil, water, plants) and animal guts, representing a significant component of the gut microbiota. The genus *Enterococcus* includes more than 20 known species, each exerting varying influences on the gut, with *Enterococcus faecalis* and *Enterococcus faecium* being the most common. *Enterococcus faecium* has been shown to enhance intestinal barrier function by reducing the secretion of inflammatory cytokines, decreasing intestinal permeability, and inhibiting the adhesion of pathogens, such as *Salmonella* and *Escherichia coli* [64]. However, certain strains of enterococci (e.g., *Enterococcus durans*) can significantly alter the composition of the gut microbiota under certain conditions, leading to inflammation [65]. Therefore, the impact of *Enterococcus* on intestinal barrier function is bidirectional. *Acinetobacter*, a genus of Gram-negative, non-fermenting bacteria, represents a significant nosocomial pathogen, particularly *Acinetobacter baumannii* [66]. Furthermore, an increased relative abundance of this microorganism has been observed in patients with ulcerative colitis [67]. The relative abundance of *Bacteroides*, *Campylobacter*, *norank_o__WCHB1-41*, and *Alistipes* increased in the CF group. *Alistipes* and *Bacteroides* belong to the phylum Bacteroides, which can maintain gut health by breaking down complex plant polysaccharides to provide energy to the host [68] and fermenting carbohydrates to produce SCFAs (such as propionic acid, acetic acid, and butyric acid) [69]. In contrast, *Campylobacter* is a class of Gram-negative, aerobic bacteria that exhibit strong pathogenicity and adaptability in the host intestine. The relative abundance of *Campylobacter* is increased in patients with ulcerative colitis and Crohn’s disease and may manifest as long-term intestinal inflammation [70,71]. In the cecum, the expressions of *Rikenellaceae_RC9_gut_group*, *Ruminococcus_torques_group*, and *norank_f__Ruminococcaceae* in the CR group were upregulated. Among them, *Ruminococcus_torques_group* and *norank_f__Ruminococcaceae* belong to the phylum Firmicutes and exhibit cellulose degradation strategies within the digestive tract. These bacteria produce cellulases to hydrolyze plant fibers, subsequently accumulating SCFAs, such as butyrate [72]. This process plays a crucial role in modulating host immune responses by suppressing inflammatory reactions [73] and promoting Treg cell differentiation [74]. *Rikenellaceae_RC9_gut_group* core metabolic functions focus on the degradation of plant polysaccharides [75] and the synthesis of short-chain fatty acids [76]. The relative abundance of *Ureaplasma* and *Campylobacter* was elevated in the CF group. *Ureaplasma* is a group of microorganisms belonging to the phylum Mollicutes, which is characterized by its ability to metabolize urea, generate ammonia, and exhibit pathogenic potential and high variability [77]. *Ureaplasma* can activate the host immune system via surface lipoproteins (e.g., multi-band antigen MBA), triggering the Toll-like receptor (TLR1/2/6) signaling pathway, and promoting the release of NF-κB-mediated inflammatory cytokines (e.g., TNF-α, IL-6) [78]. The results of correlation analysis also show that LPC was significantly positively correlated with *Ureaplasma*, and it is speculated that the increase in the relative abundance of *Ureaplasma* and *Campylobacter* was related to the intestinal inflammatory infiltration of Lueyang black-bone chickens in the CF group.

The modulation of host defenses by the microbiota may primarily be mediated through the release of common intermediate agents, such as metabolites, rather than direct interactions between specific microbes and immune cells [79]. Microbial metabolites are considered messengers of the gut microbiota, given the capacity of bacteria to produce unique molecules that the host is unable to synthesize on its own, and many immune cells in the gut express receptors for these molecules [80]. Over the past two decades, the multifaceted health benefits of omega-3 polyunsaturated fatty acids have been substantiated. In particular, the synergistic effects of the long-chain eicosapentaenoic acid (EPA) and docosahexaenoic acid (DHA) found in fish oil are believed to have cardioprotective, neuroprotective, antidepressant, metabolic-protective, anti-inflammatory, and anticancer properties [81]. Although the precise molecular mechanisms underlying their biological actions remain to be fully elucidated, recent advancements suggest that omega-3 unsaturated fatty acids interact closely with the gut microbiota in a complex and multidirectional manner [82,83,84]. Roussel C et al. found that the supplementation of omega-3 polyunsaturated fatty acids (PUFAs) in a human gut microbiota system mucosal simulator directly modulated the gut microbiota, resulting in an increase in *Akkermania mucophila*, a decrease in myxolytic bacteria of Firmicutes, and a decrease in non-mucolytic *Clostridium* in the lumen [81]. At the same time, in terms of inflammation research, it has been shown that omega-3 fatty acids, such as EPA and DHA, can slow inflammation by reducing lipid synthesis, increasing fatty acid oxidation, inhibiting fat cell lipolysis, and replacing omega-6 fatty acids and arachidonic acid from phospholipids, thereby blocking the synthesis of pro-inflammatory mediators [34]. In our study, the levels of omega-3 polyunsaturated fatty acids were significantly higher in the CR group compared to the CF group among the differentially expressed metabolites. Furthermore, the morphological findings from tissue sections also indicate that the infiltration of inflammatory cells in the intestines of the CR group was less severe than in the CF group. Therefore, it can be inferred that omega-3 polyunsaturated fatty acids may mitigate intestinal inflammation by modulating the gut microbiota and inhibiting the synthesis of pro-inflammatory mediators.

Another critical function of the gut microbiota is the breakdown of primary bile acids and their subsequent conversion to secondary bile acids, which is important for gut health [39]. Bile acids are the end products of cholesterol metabolism in the liver. Bile acids are categorized into primary and secondary forms. Primary bile acids are synthesized directly by hepatocytes, whereas secondary bile acids are generated through the action of the gut microbiota, thereby modulating host metabolism [39]. Bile acids can reduce intestinal inflammation and maintain the integrity and proliferation of intestinal epithelial cells by activating Takeda G-protein-coupled receptor 5 (TGR5 receptor) [85]. Sinha SR et al., in a mouse model of colitis, also showed that supplementation with secondary bile acids partially alleviated intestinal inflammation [86]. In this study, the levels of 12-KetoCDCA and 7α-HOAd were significantly elevated in the CR group. 12-KetoCDCA, a crucial bile acid metabolite, serves as a direct precursor to the primary bile acid chenodeoxycholic acid (CDCA), which can be further metabolized into secondary bile acids, such as lithocholic acid. As a bile acid metabolite, 12-KetoCDCA can inhibit the inflammatory response by activating the TGR5 receptor and regulating the homeostasis of intestinal immune cells [87]. 7α-HOAd is a bile acid lipid molecule, produced by cholesterol oxidative metabolism, and is involved in intermediate reactions in the bile acid synthesis pathway. The metabolic pathways of 7α-HOAd in the intestine are primarily dependent on the enzymatic activity of the gut microbiota. Furthermore, it holds significant biomedical importance as a potential biomarker for hepatobiliary diseases (e.g., cirrhosis and cholestasis), with its concentration in urine or plasma reflecting the severity of the disease [88].

The Lueyang black-bone chicken, a native breed from Lueyang County in Shaanxi Province, is recognized for its superior meat quality [89]. The black chicken’s strong foraging ability, eating habits, and wildness render free-range husbandry the most prevalent rearing method. Several studies have shown that free-range poultry generally has more tender, juicy meat, lower fat content, higher intermuscular fat content, and a better taste [90,91]. In addition, free-range chickens enjoy more opportunities for natural behaviors, such as walking, running, foraging, and dust bathing, which contribute to their welfare [92]. However, free-range chickens also face a higher risk of disease infection and mortality due to their exposure to the natural environment [93]. Our findings also indicate that the Lueyang black-bone chickens raised in cage-free conditions exhibited more pronounced intestinal inflammatory infections. Therefore, in the actual breeding, it is necessary to comprehensively consider the pros and cons of the cage-raised and cage-free modes and ensure animal health, welfare, and food quality through the scientific rearing management of Lueyang black-bone chicken to further improve their breeding efficiency and market competitiveness.

## 5. Conclusions

In summary, the present study was conducted to investigate the effects of cage-raised and cage-free rearing on the intestinal morphology, intestinal metabolites, and gut microbiota of Lueyang black-bone chickens. It was found that the degree of intestinal inflammatory cell infiltration in Lueyang black-bone chickens was slowed down in cage rearing compared with cage-free rearing, and analysis of non-targeted metabolomics data showed that the levels of omega-3 polyunsaturated fatty acids in the CR group were significantly higher than those in the CF group. The contents of 12-KetoCDCA and 7Alpha-Hydroxy-3-Oxochol-4-En-24-Oic Acid in the CR group were also increased. In contrast, the LPC and PE levels were significantly higher in the intestines of the chickens under cage-free conditions, thus promoting inflammatory responses. The results of correlation analysis also show that the content of LPC was positively correlated with *Ureaplasma*, *Bradyrhizobium*, *Brevundimonas*, *Microbacterium*, *Pelomonas*, *phyllobacterium*, *Aquabacterium*, and *Rothia*. The polyunsaturated fatty acids of the omega-3 series were positively correlated with *Flavobacterium*, *Ralstonia*, and *Caulobacter*, and the bile acid metabolites were significantly positively correlated with *Rikenellaceae_RC9_gut_group*. Further research is required to elucidate the mechanisms by which these key metabolites and microorganisms modulate intestinal immune responses and gut health, thereby providing insights for the breeding of indigenous chicken breeds.

## Data Availability

The data are contained within the article.
